# Raman spectroscopic evaluation of human serum using metal plate and 785- and 1064-nm excitation lasers

**DOI:** 10.1371/journal.pone.0211986

**Published:** 2019-02-15

**Authors:** Hiroaki Ito, Naoyuki Uragami, Tomokazu Miyazaki, Noboru Yokoyama, Haruhiro Inoue

**Affiliations:** 1 Digestive Disease Center, Showa University Koto Toyosu Hospital, Toyosu, Koto-ku, Tokyo, Japan; 2 Research Planning Department, JSR Corporation, Higashi-Sinbashi, Minato-ku, Tokyo, Japan; Institute of Materials Science, GERMANY

## Abstract

In this study, we utilized a stainless steel (SUS304) plate for measuring the Raman scattering spectra of body fluid samples. Using this stainless steel plate, we recorded the Raman scattering spectra of 99.5% ethanol and human serum samples by performing irradiation with 785- and 1064-nm lasers. Raman scattering spectra with intensities equal to or greater than those reported previously were obtained. In addition, the Raman scattering spectra acquired using the 1064-nm laser were less influenced by autofluorescence than those obtained via use of the shorter-wavelength laser. Moreover, the shapes of the spectra did not show any dependence on integration time, and denaturation of the samples was minimal. Our method, based on 1064-nm laser and the stainless steel plate, provides performance equal to or better than the methods reported thus far for the measurement of Raman scattering spectra from liquid samples. This method can be employed to rapidly evaluate the components of serum in liquid form without using surface-enhanced Raman scattering.

## Introduction

Raman scattering spectroscopy is a nondestructive evaluation method that can be used to investigate the component ratios and structures of materials in a noncontact manner [1]. The evaluated substance can be a solid [2], liquid [3], or gas [4]. Human body fluids are analyzed using Raman spectroscopy in order to gain biochemical information for medical research and several methods for obtaining the Raman scattering spectra of human body fluids have been reported [5], but standard methods have not yet been established. Because the components of human serum are diverse and exist in very small amounts, it is difficult to obtain the Raman scattering spectra of human serum that has not been pretreated without denaturing the liquid-state serum. Typically, synthetic quartz glass slides, silicone rubber o-rings, and glass coverslips are used to measure the Raman scattering spectra of liquid samples, by placing a silicone rubber o-ring on a synthetic quartz glass slide, dropping the liquid sample onto the slide, and covering it with a glass coverslip. Autofluorescence is generated if ordinary glass is used for the slide glass, so synthetic quartz, which has weaker autofluorescence, is used; however, synthetic quartz is disadvantageous because it is relatively expensive [6]. In addition, it is necessary to perform the cumbersome operation manually. In fact, the measurement results obtained using this method are often inconsistent. Surface-enhanced Raman scattering (SERS) is a phenomenon in which the intensity of the Raman scattered light of a substance adsorbed on a metal nanoparticle surface is greatly amplified by the resonance effect of localized surface plasmons [7]. While SERS spectroscopy has been used as a high-sensitivity technique for detecting trace amounts of substances [8], it is generally complicated and expensive [9].

To establish a standard method for easily and stably acquiring the Raman scattering spectra of body fluid samples, we utilized a stainless steel plate. SUS304 (18Cr-8Ni) is a steel alloy containing very small amounts of manganese, silicon, carbon, phosphorus, and sulfur, in addition to chromium and nickel. SUS304 has excellent corrosion resistance and durability among the various types of stainless steel [10]. It was reported that Raman scattering spectra with high signal-to-noise ratios were acquired from tissue and cell specimens on grade 304 super mirror stainless steel (UNS S30400) [11]. In addition, SUS304 can be shaped, so we chose it as the plate material. In this study, we measured the Raman scattering spectrum of ethyl alcohol to evaluate the performance of our stainless steel plate method by comparing it with a show any dependence conventional method for measuring liquid samples. Then, the Raman scattering spectra of human serum samples were measured.

## Materials and methods

### Sample preparation

Ethanol (99.5%) for molecular biology (Fujifilm Wako Pure Chemical Corporation, Tokyo, Japan) was used as a control in this study.

For the human samples, 10 mL of peripheral blood samples were obtained from five patients with benign disease before treatment. All of the patients underwent surgical treatment at Showa University Koto Toyosu Hospital and provided informed consent for the use of their samples in this study. The collected peripheral blood samples were deposited in Paxgene Blood ccfDNA Tubes (Preanalytix GmbH, Hombrechtikon, Switzerland). Serum samples were extracted after centrifugation of the blood samples for 15 min (1900 × g, 4°C). The extracted serum samples were placed in 1.5-mL polypropylene round-bottom microtubes (catalog number 131-715C, Fukae Kasei Co., Ltd., Kobe, Japan) and were preserved at -80°C in an ultra-low-temperature freezer (MDF-C8V1, Panasonic Corporation, Osaka, Japan).

The Institutional Review Board of Showa University approved the study. We explained the study protocol to the patients before they provided written informed consent. This study was registered with the University Hospital Medical Information Network in Japan, no. UMIN000017045.

### Raman spectroscopy

All the samples were analyzed using a Nomadic Raman microscope with a computer-controlled electronic stage (BaySpec, Inc., San Jose CA, USA), using excitation lasers with wavelengths of 785-nm and 1064-nm and a 2048×64 pixel thermoelectric cooled charge-coupled device (CCD) detector and grating unit with a spectral range of 100–3200 cm^-1^ (grating resolution 2 cm^-1^), and Pathologic System Software Version 1.0.1.0 (BaySpec, Inc.). The power of the 785-nm laser was set to 50 mW. For ethanol (99.5%), three sets of measurements were performed with 1 s integration time, and the average was taken as the measured spectrum. For the clinical serum samples, three sets of measurements were performed with the 1064-nm laser for up to 30 s and with the 785-nm laser for up to 60 s, and the average was taken as the measured spectrum in each case. From the measured spectra, the dark background noise of the CCD camera and the spectra without the sample recorded in advance were subtracted. Baseline correction was performed for each spectrum by employing the Pathologic System Software.

The spectra were collected by using a 20× magnifying objective lens with a correction collar for near-infrared microscopy (LCPLN20XIR, Olympus Corporation, Tokyo, Japan). A CCD camera (1392×1040 color CCD, up to 30 fps, Lw135R, Lumenera Corporation, Capella Court, Ottawa, ON, Canada) was used for focusing before each Raman scattering spectrum was acquired. The figures were created by utilizing JMP Pro 14.0.0 (SAS Institute, Cary, NC, USA), RaspWin Ver 8.0.1 (HT SoftLab) and Adobe Illustrator CS6 Version 16.0.3 (Adobe Systems Incorporated, San Jose CA, USA).

### Slide glass

**Fig 1:**
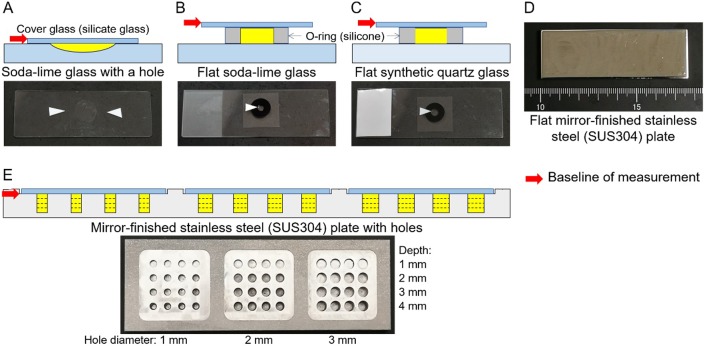
(a) Soda-lime glass with a hole, (b) flat soda-lime glass, (c) flat synthetic quartz glass, (d) flat mirror-finished stainless steel (SUS304) plate, and (e) designed mirror-finished stainless steel (SUS304) plate with holes for obtaining Raman scattering spectra of liquid samples.

We measured the samples by using a slide glass with holes (soda-lime glass, 76×26 mm, thickness 1.3 mm, hole diameter 14–15 mm, hole depth 0.6 mm, Toshin Riko Co., Ltd., Tokyo, Japan) with a cover glass (silicate glass, Micro Cover Glass, No. 1, 18×18 mm, thickness 0.12–0.17 mm, Matsunami Glass Ind., Ltd., Osaka, Japan) (Fig 1A), a normal slide glass (soda-lime glass, S1111, 76×26 mm, thickness 0.8–1.0 mm, Matsunami Glass Ind., Ltd., Osaka, Japan) with a silicone rubber o-ring (inner diameter 4 mm, thickness 1.5 mm) and cover glass (Fig 1B), and a synthetic quartz slide glass (76×26 mm, thickness 1 mm, Daico Mfg. Co., Ltd., Kyoto, Japan) with a silicone rubber o-ring and cover glass (Fig 1C). The samples were dropped onto the slide so that they just filled the space bounded by the slide glass, silicone rubber o-ring, and cover glass, with care being taken not to form bubbles.

### Stainless steel plate

We designed two types of stainless steel (SUS304) plates (76×26×5 mm). One was a mirror-finished flat stainless steel (SUS304) plate placed under the slide glass to reflect the laser light from beneath the slide glass (Fig 1D); the other was a plate containing 48 cylindrical wells (diameters 2-, 2.5-, and 3-mm and depths 1, 2, 3, and 4 mm) for Raman spectroscopy measurement of the liquid samples. Since Raman back-scattered light is more intense than the corresponding forward-scattered light, we anticipated the occurrence of Raman scattered light due to the reflection of the laser light from the bottom surfaces of the wells and we therefore performed mirror processing of the bottom of each well (Yama Techno Innovate Co., Ltd., Fukura, Oyama, Tochigi, Japan). The samples were dropped onto the slide so that they just filled the space bounded by the plate and cover glass, taking care to ensure that no bubbles were formed (Fig 1E).

## Results

### Raman scattering spectra of ethanol (99.5%) obtained using a slide glass with a hole

**Fig 2:**
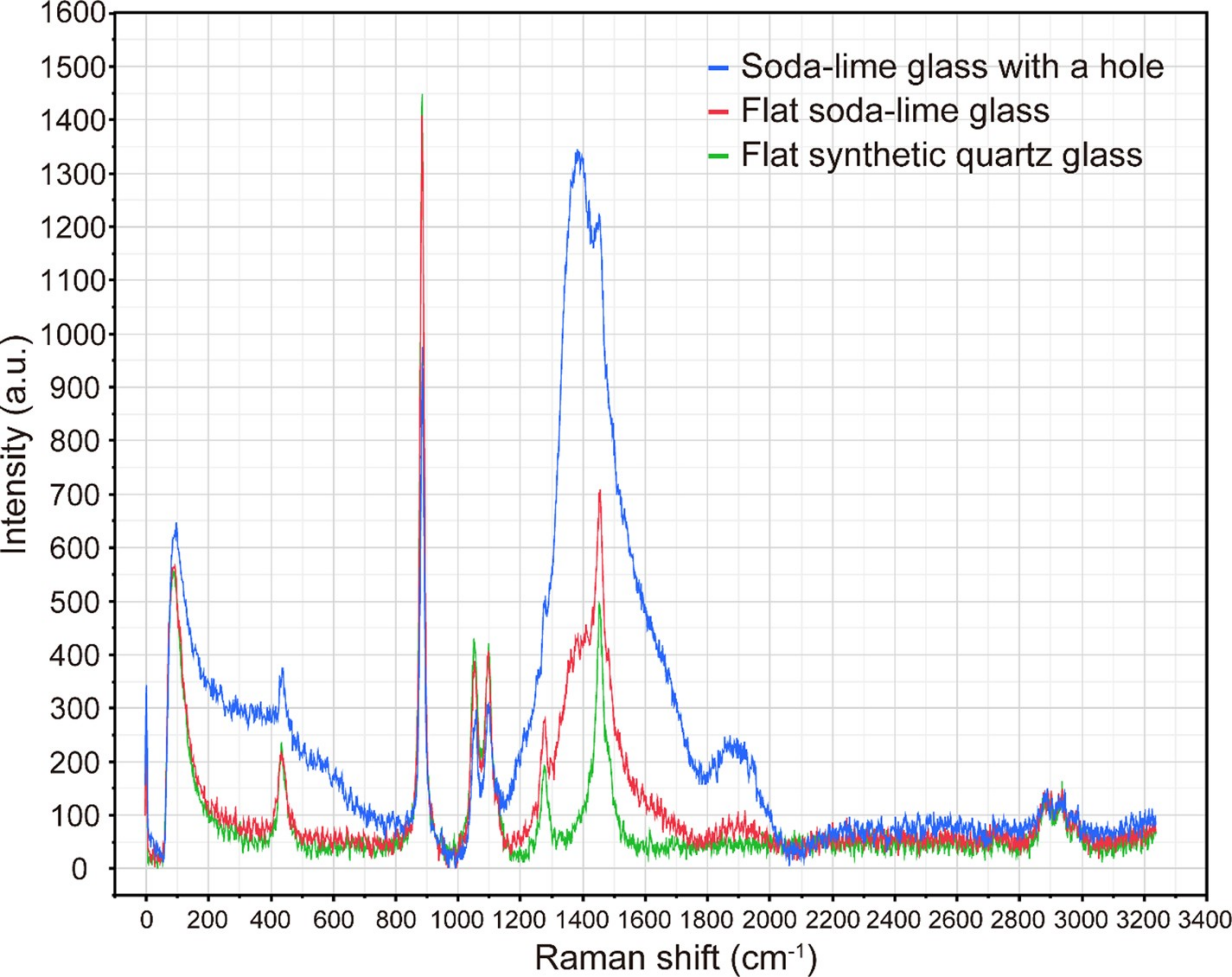
Raman scattering spectra of ethanol (99.5%) obtained using a 785-nm laser and substrates consisting of soda-lime glass with a hole, flat soda-lime glass, and flat synthetic quartz glass. Each sample was irradiated by a 785-nm laser at an output power of 50 mW for 1 s, and the average of three recorded spectra was taken as the measured spectrum. The focal positions of the laser for the soda-lime glass with a hole, flat soda-lime glass, and flat synthetic quartz glass slide were respectively set to 300, 700, and 700 μm from the lower surface of the cover glass.

**Fig 3:**
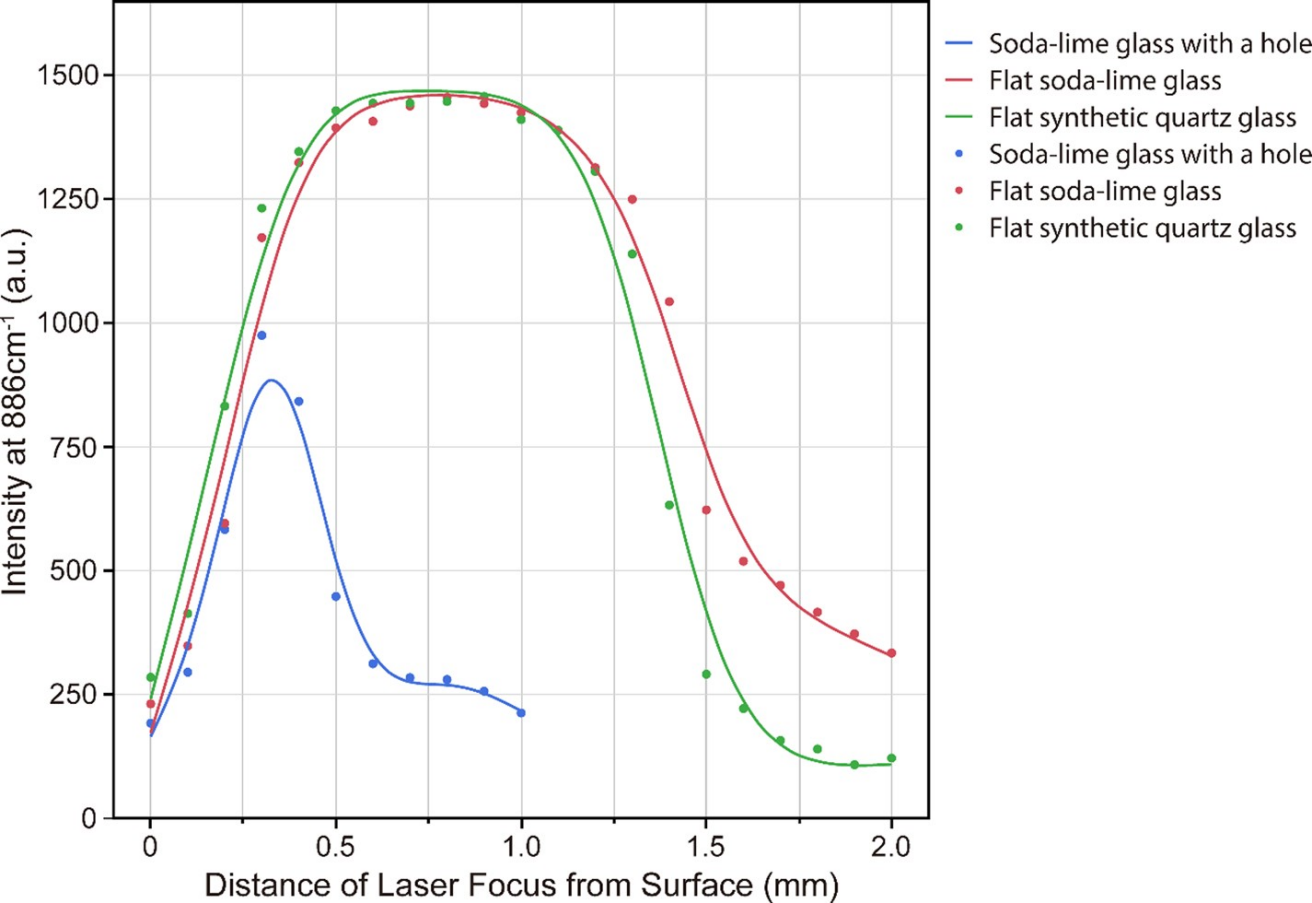
Raman scattering intensity at 886 cm^-1^ of ethanol (99.5%) obtained employing a 785-nm laser on soda-lime glass with a hole, flat soda-lime glass, and flat synthetic quartz glass substrates. The focal position of the laser in the soda-lime glass with a hole, flat soda-lime glass, and flat synthetic quartz glass cases was shifted in increments of 100 μm from the lower surface of the cover glass to depths of 1, 2, and 3 mm, respectively. For each focal position, irradiation was performed with the 785-nm laser at an output power of 50 mW for 1 s, and the average of three recorded spectra was taken as the measured spectrum. The Poisson distribution with *λ* value of the smoothed line of each measured value was set to 0.01.

We used a 785-nm laser, set the laser power to 50 mW and the integration time to 1 s, and took the average of three recorded spectra as the measured spectrum. However, in the detected Raman scattering spectra of ethanol (99.5%) including in the region of the bands corresponding to the intramolecular vibrations of C−C molecules at 886 cm^-1^, the autofluorescence from the glass was relatively strong (Fig 2) [12]. The intensity at 886 cm^-1^ in the Raman scattering spectra was strongest when the laser focal point was 300 μm from the surface of the cover glass (Fig 3).

### Raman scattering spectra of ethanol (99.5%) obtained using a conventional slide glass

We used a 785-nm laser, set the laser power to 50 mW and the integration time to 1 s, and took the average of three recorded spectra as the measured spectrum. In the collected Raman scattering spectra of ethanol (99.5%), the autofluorescence from the glass was less intense than those from the slide glass with a hole (Fig 2). The intensity at 886 cm^-1^ in the Raman scattering spectrum was the strongest at a point 700 μm from the lower surface of the cover glass (Fig 3).

### Raman scattering spectra of ethanol (99.5%) obtained using a synthetic quartz slide glass

We used a 785-nm laser, set the laser power to 50 mW and the integration time to 1 s, and took the average of three recorded spectra as the measured spectrum. The measured Raman scattering spectra of ethanol (99.5%) included only a weak autofluorescence contribution from the glass (Fig 2). The intensity at 886 cm^-1^ in the Raman scattering spectrum was the strongest at a point 700 μm from the lower surface of the cover glass (Fig 3).

**Fig 4:**
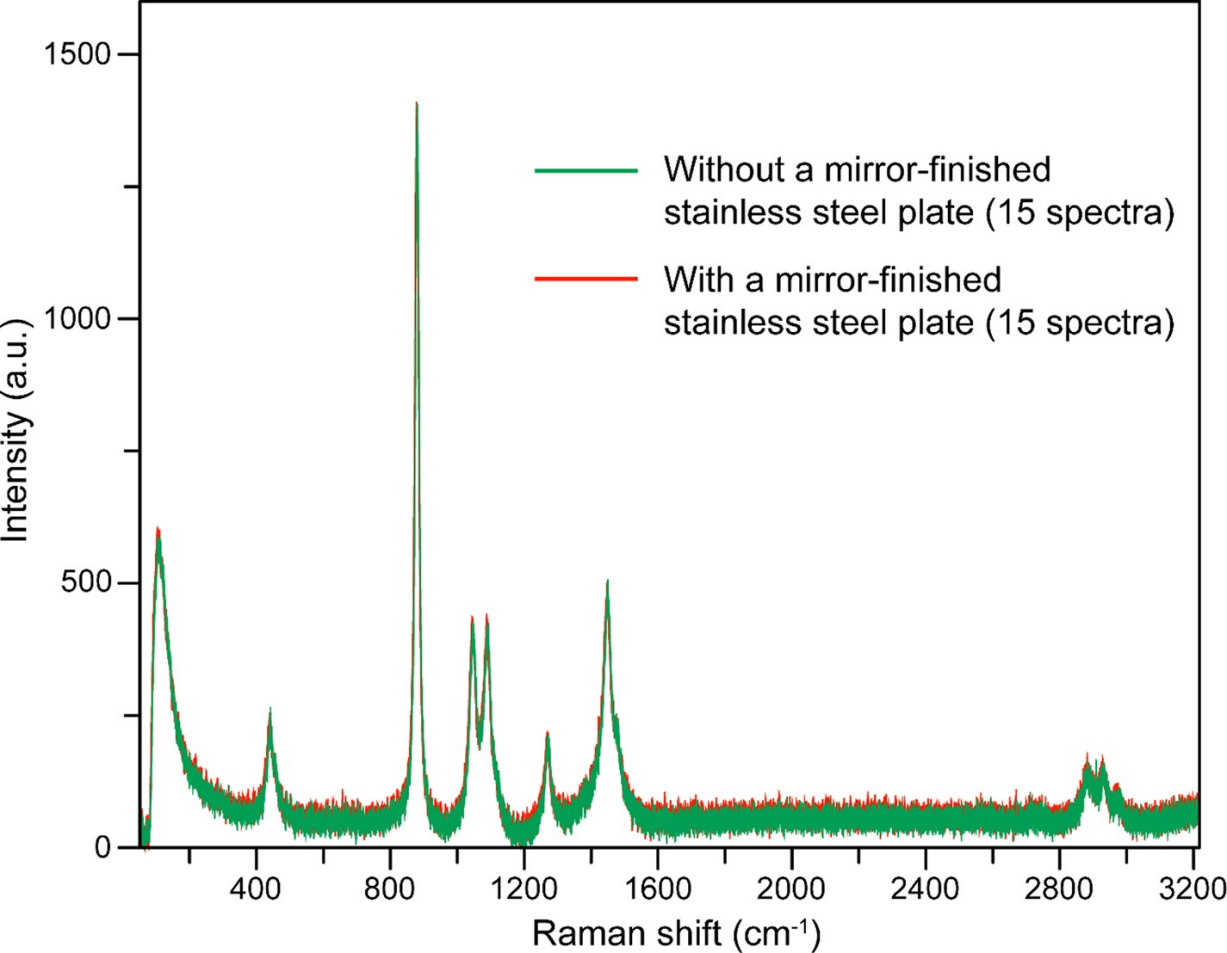
Raman scattering spectra of ethanol (99.5%) obtained using a 785-nm laser and flat synthetic quartz glass with or without a flat mirror-finished stainless steel plate as the substrate. For each focal position, irradiation was performed with the 785-nm laser at an output power of 50 mW for 1 s, and the average of three recorded spectra was taken as the measured spectrum. The focal position of the laser was set to a depth of 700 μm from the lower surface of the cover glass. Fifteen spectra recorded using only flat synthetic quartz glass and 15 spectra recorded with a flat mirror-finished stainless steel plate under the flat synthetic quartz glass are compared.

To confirm the influence of the Raman back-scattered light, a mirror-finished flat stainless steel plate was placed under the synthetic quartz glass slide, and the irradiated laser was reflected from it [6]. Then, Raman measurements were performed under the same conditions. No difference was observed between the 15 spectra obtained with and without the mirror-finished flat stainless steel plate under the synthetic quartz glass slide (Fig 4).

### Raman scattering spectra of ethanol (99.5%) obtained using a mirror-finished stainless steel (SUS304) plate with wells

**Fig 5:**
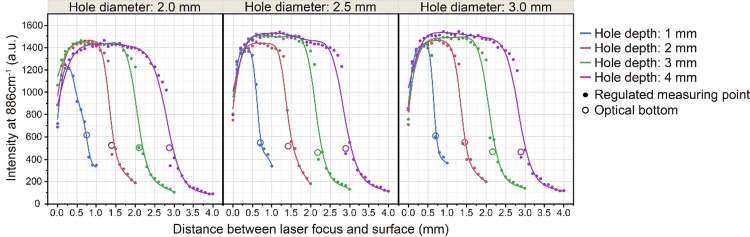
Relationships among well diameter, laser focal point, and Raman scattering intensity at 886 cm^-1^ for ethanol (99.5%) obtained using a 785-nm laser and a stainless steel plate with wells. A total of 12 different well sizes were employed, with diameters of 2-, 2.5-, and 3-mm and depths of 1, 2, 3, and 4 mm. The laser focal position was shifted in increments of 100 μm from the surface of the plate to the bottom of the well. For each focal position, irradiation was performed with the 785-nm laser at an output power of 50 mW for 1 s, and the average of three recorded spectra was taken as the measured spectrum. The *λ* value of the smoothed line of each measured value was set to 0.01.

**Fig 6:**
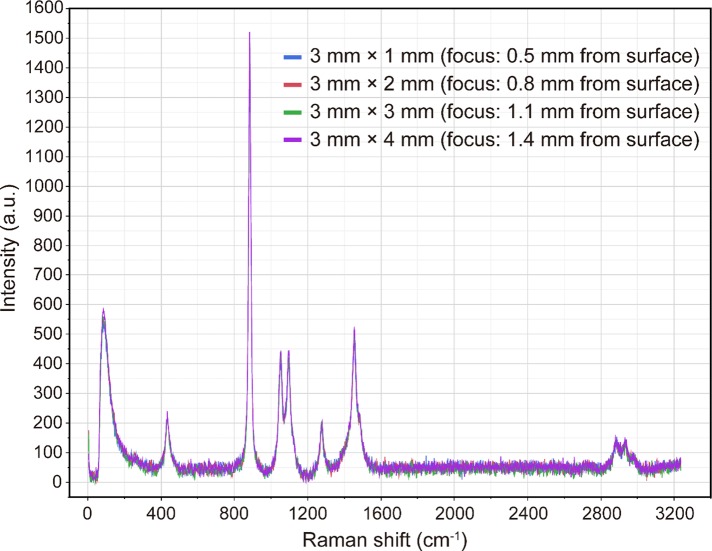
Raman scattering spectra of ethanol (99.5%) obtained using a 785-nm laser and a stainless steel plate with wells. For each focal position, irradiation was performed with the 785-nm laser at an output power of 50 mW for 1 s, and the average of three recorded spectra was taken as the measured spectrum. The well diameter was 3 mm in each case, while the well depth was set to 1, 2, 3, and 4 mm and the laser focus was set to 500, 800, 1100, and 1400 μm, respectively, from the surface of the plate.

We used a 785-nm laser, set the laser power to 50 mW and the integration time to 1 s, and took the average of three recorded spectra as the measured spectrum. The measured Raman scattering spectra of ethanol (99.5%) included only a weak autofluorescence contribution from the mirror-finished stainless steel with wells. To obtain a greater Raman scattering intensity, it was necessary to focus the laser at a particular distance from both the cover glass and the bottom of the well. The laser focus had to be separated from the lower surface of the cover glass by at least 0.2 mm. The optimum distance from the laser focus to the stainless steel surface beneath was not affected by the well diameter, but it depended on the well depth and needed to be at least 0.5, 0.7, 1.0, and 1.3 mm for well depths of 1, 2, 3, and 4 mm, respectively. These optimal distances were presumed to be related not to the physical distance from the SUS304 stainless steel plate surface to the bottom, but to the distance between the optical focus and the bottom (Fig 5). For the 2-, 2.5-, and 3-mm-diameter wells, if the laser focal depth was properly set, quite similar Raman scattering spectra were obtained, and these were very similar to the spectra acquired using a synthetic quartz glass slide (Fig 6).

**Fig 7:**
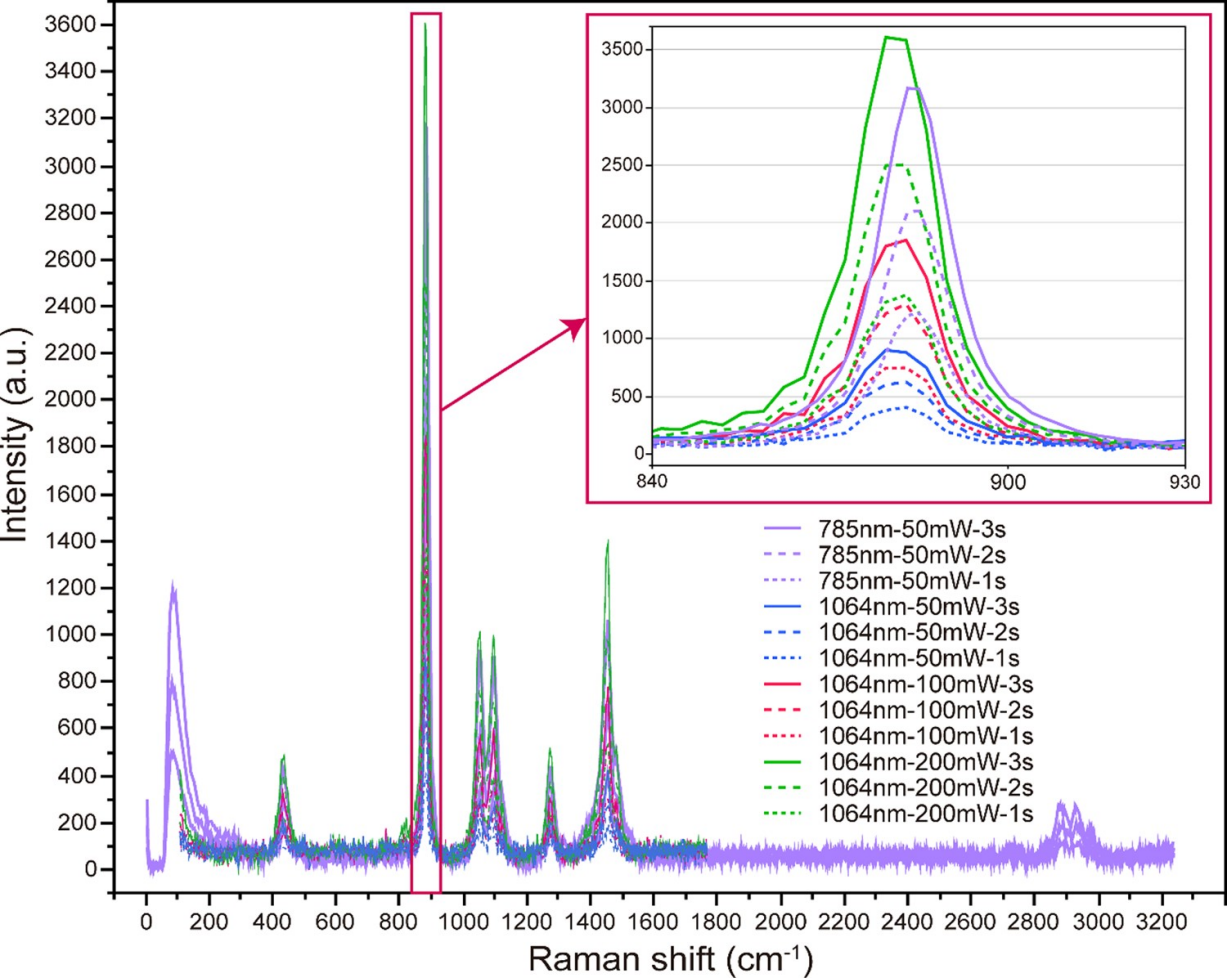
Raman scattering spectra of ethanol (99.5%) obtained employing 785- and 1064-nm lasers and a stainless steel plate with a well. Using a 3-mm-diameter, 4-mm-deep well, the laser focal position was set at 1500 μm from the surface of the plate. The 785-nm laser irradiation was performed for 1, 2, and 3 s at an output power of 50 mW. The 1064-nm laser irradiation was performed for 1, 2, and 3 s at output powers of 50, 100, and 200 mW. The average of three recorded spectra was taken as the measured spectrum.

Next, we used a 1064-nm laser, changed the laser power from 50 mW to 200 mW, and varied the integration time from 1 to 3 s. Raman scattering spectra with significant intensities were obtained under each set of conditions, and the scattering intensities were correlated with both the laser output and integration time (Fig 7). The Raman scattering intensity at 886 cm^-1^ obtained by performing irradiation with the 1064-nm, 200-mW laser for 3 s was the strongest, and that obtained by performing irradiation with the 785-nm, 50-mW laser for 3 s was lower by about 450 a.u. (Fig 7).

### Raman scattering spectra of clinical serum samples obtained using a mirror-finished stainless steel (SUS304) plate

The Raman scattering spectra of human serum were measured using a stainless steel (SUS304) plate having wells with mirror finish at the bottom of each well. We used a maximum diameter of 3 mm and depth of 4 mm to avoid sample denaturation by laser irradiation.

**Fig 8:**
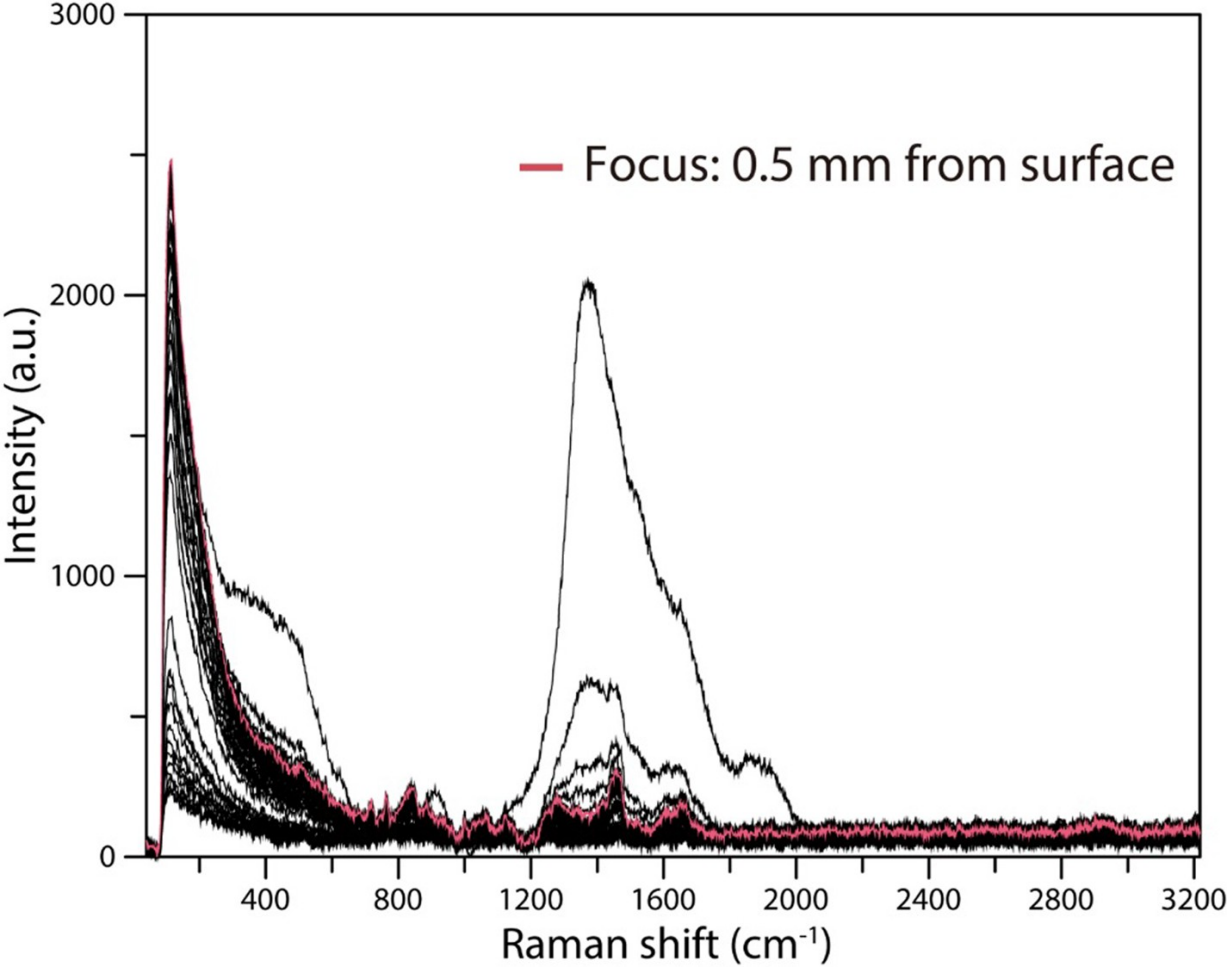
Relationship between laser focal point and Raman scattering spectra of human serum obtained utilizing a 785-nm laser and a stainless steel plate with a well. Using a 3-mm-diameter, 4-mm-deep well, 785-nm laser irradiation was performed for 5 s at an output power of 50 mW. The measurements were performed while shifting the focal position of the laser from 0 to 4 mm from the surface in increments of 100 μm.

We initially employed a 785-nm laser, but a significant spectrum could not be obtained by performing measurements with the laser focus at 1.5 mm, as determined from the ethanol measurement results. Therefore, to confirm the optimum laser focal position with respect to the serum, the laser focal position was shifted from 0 to 4 mm in 0.1-mm intervals, and the Raman scattering spectrum was recorded at each position. To obtain reliable Raman scattering spectra, we set the laser power to 50 mW and the integration time to 5 s. The results revealed that the spectrum obtained with the laser focus at 0.5 mm had the best signal-to-noise ratio (Fig 8). Therefore, we determined the optimal laser focal depth for Raman scattering spectrum measurements of serum to be 0.5 mm.

**Fig 9:**
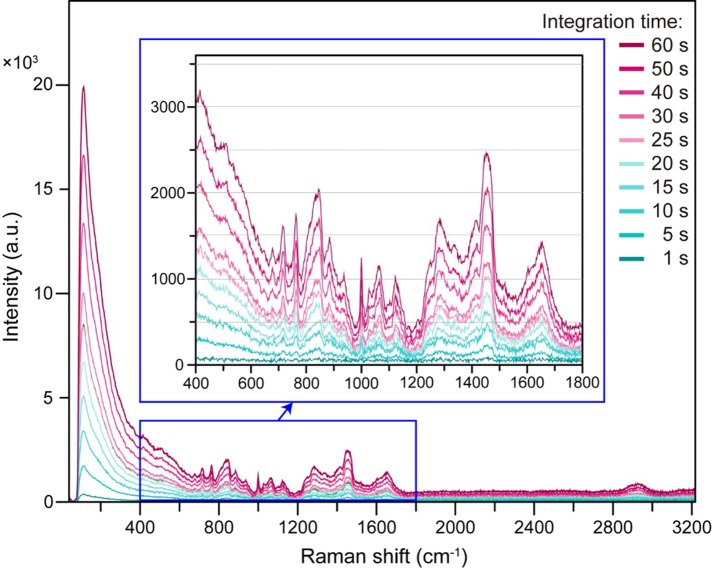
Relationship between integration time and Raman scattering spectra of human serum obtained employing a 785-nm laser and a stainless steel plate with a well. Using a 3-mm-diameter, 4-mm-deep well, 785-nm laser irradiation was performed at an output power of 50 mW. The focal position of the laser was set to a depth of 500 μm from the surface. The measurements were conducted while varying the integration time from 1 to 60 s.

We subsequently recorded the Raman scattering spectra while changing the integration time from 1 s to 60 s and observed that the Raman scattering intensity increased with increasing integration time. The measured autofluorescence background to the Raman spectrum was strong, and no significant peaks corresponding to wavelengths shorter than Raman shifts of 800 cm^-1^ could be observed (Fig 9).

**Fig 10:**
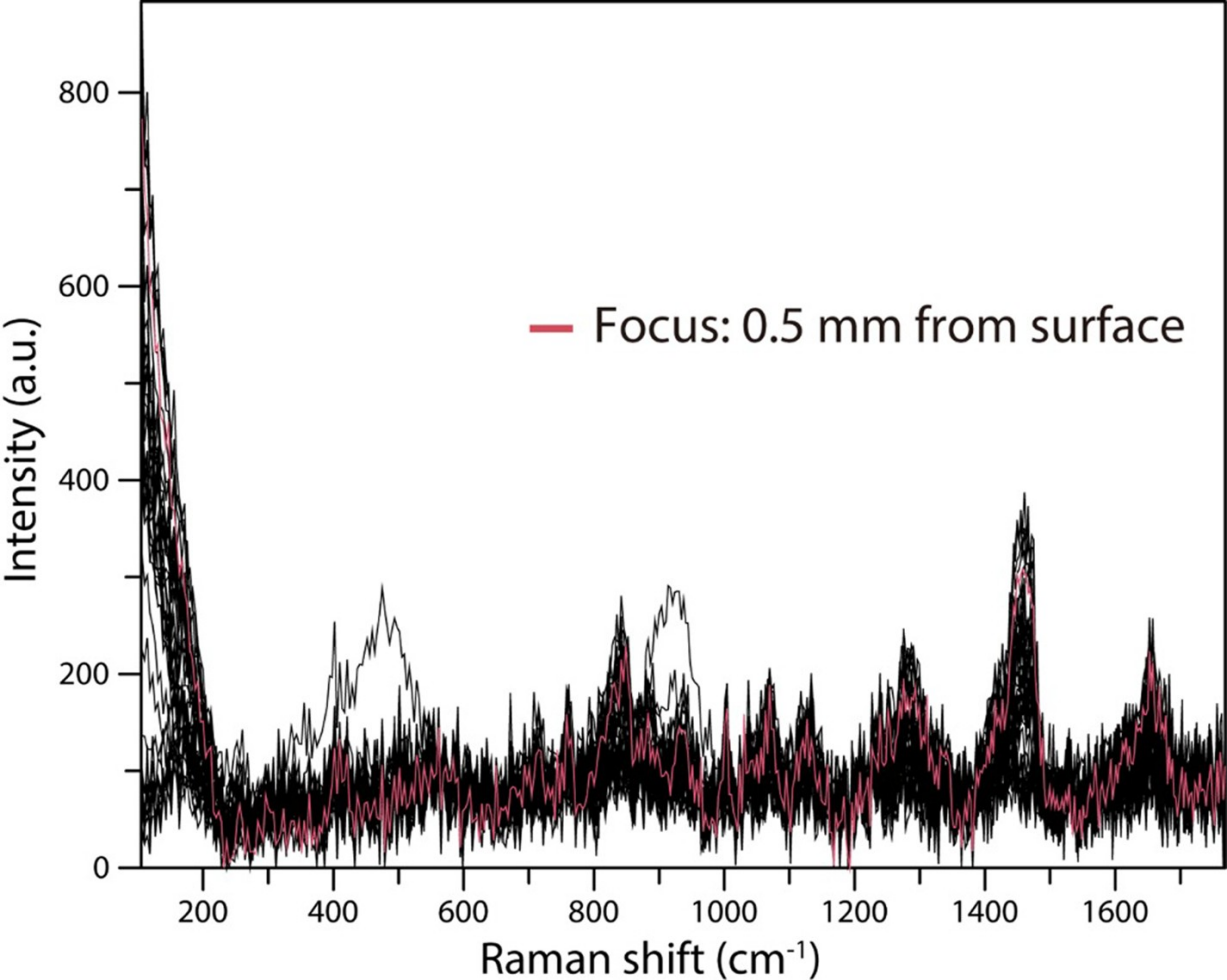
Relationship between laser focal point and Raman scattering spectra of human serum obtained employing a 1064-nm laser and a stainless steel plate with a well. Using a 3-mm-diameter, 4-mm-deep well, 1064-nm laser irradiation was performed for 5 s at an output power of 200 mW. The measurements were performed while shifting the focal position of the laser from 0 to 4 mm below the surface in increments of 100 μm.

**Fig 11:**
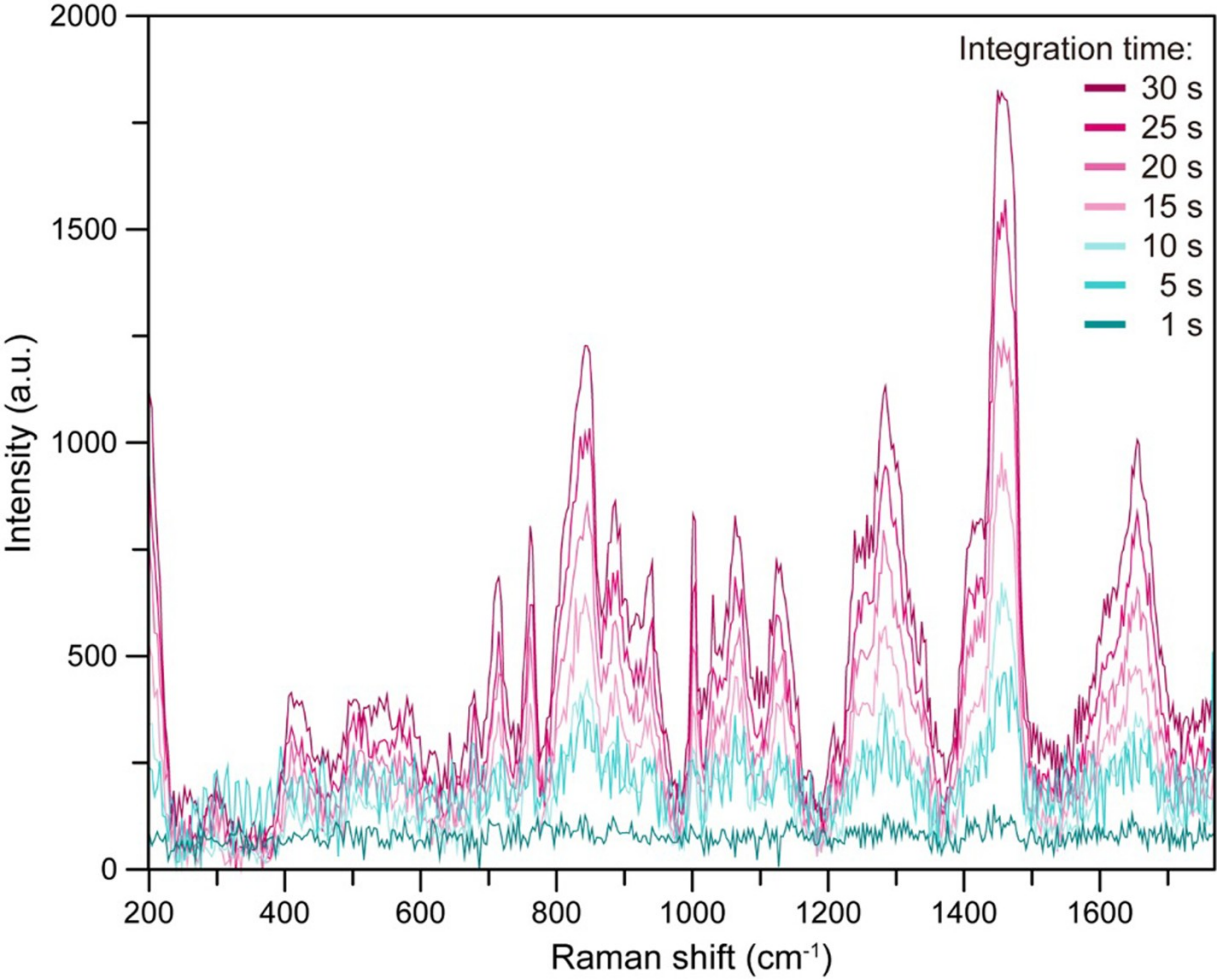
Relationship between integration time and Raman scattering spectra of human serum sample obtained employing a 1064-nm laser and a stainless steel plate with a well. Using a 3-mm-diameter, 4-mm- deep well, 1064-nm laser irradiation was performed at an output power of 200 mW. The focal position of the laser was set to a depth of 500 μm below the surface. The measurements were conducted while varying the integration time from 1 to 30 s.

We then performed measurements with both the 1064-nm and 785-nm lasers. We set the laser power to 200 mW and changed the laser focal depth. The optimum laser focal depth was determined to be 0.5 mm, the same as that obtained when only the 785-nm laser was employed (Fig 10). While changing the integration time from 1 to 30 s, the intensity of the Raman scattering increased with increasing integration time. Since the autofluorescence due to the 1064-nm laser was less intense than that generated by the 785-nm laser, it was possible to detect significant peaks at Raman shifts of less than 240 cm^-1^ (Fig 11).

## Discussion

Raman scattering spectroscopy is a non-destructive inspection method involving a laser light source and can be used to analyze the components and structures of gases, liquids, and solids. When the substance to be evaluated exists in a very small amount, it is difficult to obtain a Raman scattering spectrum with a significant signal-to-noise ratio. There are only very small amounts of disease-related substances to be detected in serum [13]. Therefore, to obtain a high Raman scattering intensity, the output power of the laser used for irradiation or the laser irradiation time is generally increased. However, human serum is a fragile substance whose properties can easily change upon heating [14]. Therefore, it is necessary to reduce the laser output power and irradiation time as much as possible. To detect substances with low concentrations in liquid samples via Raman spectroscopy, the samples are frequently dried before measurement. Correspondingly, techniques for analyzing dry serum by performing Raman scattering spectroscopy have been reported [15] However, since the substances in serum may become denatured by drying, we wanted to analyze serum in its original liquid state [16]. SERS is used to detect trace amounts of substances with high sensitivity, but the instability of the enhancement and the complexity and high cost of this method are significant drawbacks [17]. Therefore, we decided not to use SERS in this study because we wanted to develop a stable and sensitive quantitative technique for the detection of trace-substances in serum. If a sample is in a stable state before laser irradiation, the laser can be accurately irradiated at the target point, which allows the laser output power and irradiation time to be reduced as much as possible, and sample degeneration can be minimized. Therefore, in this study, we fabricated a device for Raman spectroscopic analysis of very fragile samples of human body fluids, including serum, that causes less damage.

Considering the balance between cost and effectiveness, we selected chemically and physically stable SUS304 stainless steel as a material. SUS304 can also be shaped and is not usually magnetic. Therefore, we determined that SUS304 was suitable for our purposes. Pure metals do not emit Raman scattered light, but there was the possibility of SUS304 emitting Raman scattering spectra. Therefore, before we fabricated the plates, we irradiated a piece of SUS304 with a laser with the aim of confirming the presence or absence of a Raman scattering spectrum, and no clear Raman scattering spectrum was observed. Regarding the placement of the sample, we had little information in advance about which conditions would be optimal, so we formed cylindrical wells of various sizes in the SUS304 plate.

To evaluate the prepared plate, we employed ethanol as a test sample and recorded its Raman scattering spectra. It was possible to obtain Raman scattering spectra of ethanol (99.5%) similar to those acquired when synthetic quartz glass is used, regardless of the size of the cylindrical well, by performing irradiation with a 785-nm laser. However, when the laser focus was close to the cover glass, the Raman scattering spectrum or autofluorescence of the coverslip glass was detected, and acquisition of the Raman scattering spectrum of ethanol was hindered. In addition, when the laser focus was close to the bottom of the well, the Raman scattering intensity of ethanol was low. Therefore, the depth of the well in which the sample is placed should be take a particular value, for example, 2 mm or more, to obtain more stable Raman scattering spectra. With regard to the sample damage caused by the laser irradiation, this is considered to be small, especially for larger amounts of the sample. Therefore, a 3-mm-diameter, 4-mm-deep well was designated as being the most suitable for Raman scattering spectroscopic analysis of liquid samples using our plate. We assumed that Raman back-scattered light, i.e., the light scattered towards the detector, was generated by the laser light reflected from the bottom of the well in the plate, and it was presumed that the Raman scattered signal would be enhanced by this reflection. Therefore, we added a mirror finish to the bottom of the well. However, even when a mirror-finished flat stainless steel plate was placed under a synthetic quartz slide glass slide, the Raman scattering intensity was not enhanced and the spectral pattern did not change compared with the case without the flat stainless steel plate. However, since the flatness of the bottom surface of the well was improved by mirror finishing, this finishing increased the precision of the plate.

In our Raman scattering spectroscopic analysis of the serum samples, the optimum laser focal depth was shallower than that when ethanol was used. The optimum laser focal depth for the serum analysis was 0.5 mm from the surface of the stainless steel plate for both the 785- and 1064-nm lasers. Since the refractive indices of ethanol and human serum are about the same, about 1.35, it is considered that there are factors other than the refractive index, such as light absorption rate, that affect this depth [18].

Irradiation by a 1064-nm laser results in less autofluorescence than irradiation by a 785-nm laser, so we also attempted Raman scattering spectroscopy with a 1064-nm laser [19]. Observation of the Raman scattering spectra of human serum obtained with 785-nm and 1064-nm lasers revealed that the intensity of the spectra increased with increasing laser integration time. However, the autofluorescence tended to be less intense when the 1064-nm laser was used.

In summary, for Raman scattering spectroscopy of liquid samples containing human body fluids, our method based on the stainless steel plate is easier than the commonly used method involving slide glass. Furthermore, we were able to obtain clean Raman scattering spectra of human serum, with high signal-to-noise ratios and low autofluorescent background signal levels, by employing our stainless steel plate and 1064-nm excitation laser, without using SERS.

## Conclusions

The Raman scattering spectrum measurement method involving the stainless steel (SUS304) plate, provides performance equal to the methods reported so far for liquid samples. In particular, we succeeded in rapidly evaluating the components of serum in liquid form by employing 1064-nm lasers without using SERS. However, to obtain significantly intense Raman scattering spectra, it is necessary to optimize the focal position of the laser. These results will aid in the development of a general-purpose technology to carry out high-precision disease diagnosis conveniently using blood samples.

## Supporting information

S1 File Original data of Fig 2. (TXT)

S2 File Original data of Fig 3. (TXT)

S3 File Original data of Fig 4 (quartz with mirror finished stainless plate). (TXT)

S4 File Original data of Fig 4 (quartz without mirror finished stainless plate). (TXT)

S5 File Original data of Fig 5. (TXT)

S6 File Original data of Fig 6 (laser focus was set to 500 μm). (TXT)

S7 File Original data of Fig 6 (laser focus was set to 800 μm). (TXT)

S8 File Original data of Fig 6 (laser focus was set to 1100 μm). (TXT)

S9 File Original data of Fig 6 (laser focus was set to 1400 μm). (TXT)

S10 File Original data of Fig 7. (TXT)

S11 File Original data of Fig 8. (TXT)

S12 File Original data of Fig 9. (TXT)

S13 File Original data of Fig 10. (TXT)

S14 File Original data of Fig 11. (TXT)
